# Harnessing private sector strategies for family planning to deliver the Dual Prevention Pill, the first multipurpose prevention technology with pre‐exposure prophylaxis, in an expanding HIV prevention landscape

**DOI:** 10.1002/jia2.26346

**Published:** 2024-08-15

**Authors:** Catherine Verde Hashim, Emma Llewellyn, Rob Wood, Tracey Brett, Tinashe Chinyanga, Karen Webb, Kate Segal

**Affiliations:** ^1^ AVAC New York New York USA; ^2^ Genesis Analytics Nairobi Kenya; ^3^ Halcyon Consultancy Nairobi Kenya; ^4^ Private Consultant Johannesburg South Africa; ^5^ The Organization for Public Health Interventions and Development (OPHID) Harare Zimbabwe

**Keywords:** contraception, HIV, pregnancy, unplanned, pre‐exposure prophylaxis, private sector, reproductive health

## Abstract

**Introduction:**

The Dual Prevention Pill (DPP) combines oral pre‐exposure prophylaxis (PrEP) with oral contraception (OC) to prevent HIV and pregnancy. Noting the significant role played by the private sector in delivering family planning (FP) services in countries with high HIV burden, high level of private sector OC uptake, and the recent growth in self‐care and technology‐based private sector channels, we undertook qualitative research in Kenya, South Africa and Zimbabwe to prioritize private sector service delivery approaches for the introduction of the DPP.

**Methods:**

Between March 2022 and February 2023, we conducted a literature review and key informant interviews with 34 donors and implementing partners, 19 government representatives, 17 private sector organizations, 13 pharmacy and drug shop representatives, and 12 telehealth agencies to assess the feasibility of DPP introduction in private sector channels. Channels were analysed thematically based on policies, level of coordination with the public sector, data systems, supply chain, need for subsidy, scalability, sustainability and geographic coverage.

**Results:**

Wide geographic reach, ongoing pharmacy‐administered PrEP pilots in Kenya and South Africa, and over‐the‐counter OC availability in Zimbabwe make pharmacies a priority for DPP delivery, in addition to private networked clinics, already trusted for FP and HIV services. In Kenya and South Africa, newer, technology‐based channels such as e‐pharmacies, telehealth and telemedicine are prioritized as they have rapidly grown in popularity due to nationwide accessibility, convenience and privacy. Findings are limited by a lack of standardized data on service uptake in newer channels and gaps in information on commodity pricing and willingness‐to‐pay for all channels.

**Conclusions:**

The private sector provides a significant proportion of FP services in countries with high HIV burden yet is an untapped delivery source for PrEP. Offering users a range of access options for the DPP in non‐traditional channels that minimize stigma, enhance discretion and increase convenience could increase uptake and continuation. Preparing these channels for PrEP provision requires engagement with Ministries of Health and providers and further research on pricing and willingness‐to‐pay. Aligning FP and PrEP delivery to meet the needs of those who want both HIV and pregnancy prevention will facilitate integrated service delivery and eventual DPP rollout, creating a platform for the private sector introduction of multipurpose prevention technologies.

## 1 INTRODUCTION

Unmet need for HIV prevention continues to persist worldwide, particularly in Eastern and Southern Africa, where nearly 21 million people, 53% of the global total, are living with HIV, and 500,000 new acquisitions occurred in 2022 [1]. Women and girls are disproportionately impacted, accounting for 67.5% of new HIV acquisitions among adults aged 15–49 in the region, with incidence rates among adolescent girls and young women three times higher than males of the same age [2]. Women of reproductive age (WRA) in this region have an intersecting high unmet need for family planning (FP) at 15%, compared to the global average of 9% [3], though high HIV incidence has also been found among those using modern contraception in the Evidence for Contraceptive Options and HIV Outcomes (ECHO) trial in Eswatini, Kenya, South Africa and Zambia [4]. These figures underscore the urgent need to optimize access to HIV prevention and FP for women and girls in Eastern and Southern Africa.

Multipurpose prevention technologies (MPTs), products designed to address multiple health concerns, can simplify HIV and pregnancy prevention. While commercially available MPTs are currently limited to condoms, there are over two dozen MPTs in development combining antiretrovirals (ARVs) and contraception [5]. These products hold the potential to address adherence and uptake challenges seen with oral pre‐exposure prophylaxis (PrEP) and stigma associated with HIV service delivery. The Dual Prevention Pill (DPP), a daily oral pill co‐formulated with tenofovir disoproxil fumarate and emtricitabine, a form of oral PrEP, and the combined oral contraceptions (OCs) levonorgestrel and ethinyl oestradiol, is likely to be the first of these to come to market, potentially as early as 2025 [6]. Adding a new option to the HIV prevention and FP method mix could increase both the modern contraceptive prevalence rate and PrEP coverage in countries where it is rolled out [7], and therefore, represents a significant public health opportunity.

The private sector plays a substantial role in healthcare delivery in sub‐Saharan Africa, particularly for FP services. A 2015 analysis estimated that over one‐third of women using modern contraception in sub‐Saharan Africa obtained it from a private provider [8]. In addition to traditional private sector channels such as private health facilities and pharmacies, recent years have seen rapid growth in private technology‐based channels, including telemedicine [9], further accelerated by the COVID‐19 pandemic [10]. These channels offer high potential to increase access to healthcare services, with GSMA estimating that 50% of sub‐Saharan Africa's population will be covered by mobile phone connectivity by 2025 [11]. Harnessing the reach of existing and newer private sector channels will be critical to a successful rollout of the DPP and any future MPTs. Noting this, we undertook a rapid qualitative scoping exercise [12] to better understand private sector opportunities for DPP delivery and design a private sector delivery strategy for the DPP [13] that could be adapted for other MPTs. Kenya, South Africa and Zimbabwe were selected for scoping, as they are being prioritized for DPP introduction due to high HIV burden [14] moderate‐to‐high OC use (8.1% of married WRA in Kenya, [15] 7.3% of WRA in South Africa, [16] and 27% of WRA in Zimbabwe [17]); moderate‐to‐high unmet need for FP [3] and enabling environments, particularly for PrEP scale‐up [18].

## 2 METHODS

We explored opportunities for DPP delivery via the private sector using a desk review, key informant interviews (KIIs) and stakeholder consultations. Stakeholder identification was informed by Masefield et al.’s use [19] of the 7Ps of Stakeholder Engagement, which can be used to define key health stakeholder groups: patients and the public, providers, purchasers, payers, policymakers, product makers and principal investigators [20]. We also conducted our applied research in line with Alemanno's three components of effective stakeholder engagement: communication (desk review and national scoping of key stakeholders); consultation (KIIs); and participation (stakeholder feedback workshops) [21]. These methods allowed for a comprehensive scoping of the different private sector channels available and enabled us to probe the opportunities for the DPP more deeply with key stakeholders. The stakeholder consultations were intended to pre‐empt introduction challenges caused by late engagement [22] as seen with PrEP in the private sector, and enabled an active discussion of findings from the desk review and KIIs along with opportunities for linkages and connections between different channels. Data collection and analysis took place in Kenya, South Africa and Zimbabwe between March 2022 and February 2023.

### 2.1 Desk review

During the initial research stage, in March 2022, we reviewed existing publicly available peer‐reviewed and grey literature relevant to MPTs, the DPP, PrEP, FP and the role of the private sector (as defined in Table 1) in service delivery, across the three study countries and globally, to identify opportunities and gaps for the rollout of the DPP in the private sector in Kenya, South Africa and Zimbabwe. A team of seven researchers from the three countries started by reviewing AVAC‐provided materials on the DPP and PrEP in the private sector and related materials on PrEPWatch.org, and conducted further research on programmes and organizations identified during the review and those known to be delivering FP via the private sector. Scoping conversations surfaced further documents for review. A review of published literature through PubMed was carried out using keyword searches in English (Supporting Information 1), resulting in over 100 documents, web pages and reports being reviewed. The evidence identified in the review was found to be largely fragmented and out‐of‐date and failed to capture questions related to country‐specific health markets and variable private sector regulatory processes in the three countries. These evidence gaps informed the broader market‐wide approach taken in the subsequent phases of data collection and informed the development of the KII topic guide.

**Table 1:** Private sector channels evaluated

Channel	Definition
**Pharmacy**	A physical private shop or dispensary where medicinal drugs are prepared or sold to clients over the counter. The drugs and health products that a pharmacy can provide differ between countries and are dependent on national laws and regulations. Pharmacies may have formalized links with telemedicine providers so that walk‐in clients can access a clinician's services via telemedicine from within the pharmacy.
**E‐pharmacy**	A virtual shop or dispensary where drugs and products are prepared or sold online and dispensed by direct delivery to clients through various means including post, courier service, and other, newer methods such as “drop‐off lockers.” The drugs and health products that an e‐pharmacy can provide differ between countries and are dependent on national laws and regulations.
**Telehealth**	The provision of healthcare remotely by means of telecommunications technology. Includes a wide range of providers and channels that support the consultation, referral, and delivery of services and products, such as mobile apps, online training for health workers and consultations with online medical providers. Telehealth does not frequently provide a physical service or product but refers and prescribes to clients who then obtain a service elsewhere.
**Telemedicine**	A sub‐set of telehealth which focuses on use of telecommunication and information technology for the purpose of providing remote health assessments and therapeutic interventions. Some telemedicine companies dispense pharmaceutical products including FP commodities.
**Community distribution**	The distribution of health products and services by a trained community health worker. This is the most decentralized level of healthcare and the services and products that can be provided differ between countries and are dependent on national laws and regulations.
**Mobile outreach**	The physical delivery of health services and products to communities that are typically more remote and harder to reach. Mobile outreach is typically delivered by trained medical providers who move between locations, including static facilities and pop‐up clinics.
**NGO**	Not‐for‐profit organizations that provide health services through numerous channels, including hospitals and clinics, mobile outreach services, community distribution and, in some cases, provide support to other private providers such as franchised networks. Many NGOs provide health services in urban and semi‐urban areas.
**FBO**	Similar to NGOs, except their delivery channels are supported by an organization that is driven by their faith and religious beliefs. Unlike NGOs, FBOs tend to operate in rural areas where they provide essential services. Some faiths may not support certain health services to be provided within their institutions; for example, Catholic FBOs may not offer FP.
**Networked private providers**	A group of private clinicians, clinics and hospitals that make up an association or network. Such networks can be run by one company which runs several clinics or hospitals, or they can be part of private sector associations.
**Social franchises**	Private clinics that are part of a branded and franchised chain or network, typically supported by an NGO to offer some subsidized services.

### 2.2 Key informant interviews

One hundred and six KIIs were conducted between April and December 2022 with a diverse selection of private sector stakeholders, representing government bodies, donors and development partners, and private providers, including representatives from newer, technology‐based service delivery channels such as telemedicine companies and e‐pharmacies (Table 2). Interviews were carried out both remotely and in‐person in Kisumu and Nairobi, Kenya; Cape Town, Johannesburg and Pretoria, South Africa; and Bulawayo, Gwanda and Harare, Zimbabwe, by experienced FP and HIV private sector experts based in each country. All participants interviewed in person provided written informed consent, while those interviewed remotely provided verbal consent, as well as consent to be recorded. Interviews were conducted in English. Notes were taken for in‐person interviews while remote interviews were recorded, with a summary of each interview transcribed afterwards.

**Table 2:** Private sector stakeholders consulted

	Number of organizations consulted^a^		
Stakeholder category	Kenya	South Africa	Zimbabwe
National and sub‐national governments, MoH, regulatory bodies, and logistics and supply bodies	11	6	2
Donors, development partners and implementing agencies	7	9	3
Private sector organizations, networks and associations	4	6	7
Pharmacies and drug shops	4	4	5
Telehealth	6	5	1
NGOs and FBOs	4	4	7
Total	**36**	**34**	**25**

^a^In some instances, more than one stakeholder was consulted at an organization, or a follow‐up interview was done.

Semi‐structured interviews were conducted according to three separate topic guides tailored to stakeholder type. Donors and development partners were asked about their country's PrEP landscape, opportunities and barriers for PrEP and OC delivery, the role of the private sector, opportunities and challenges for potential DPP rollout, available PrEP market data and PrEP financing. The topic guide for government representatives covered the same themes, in addition to PrEP and OC supply chains, the policy and regulatory environment, and partnerships and technical working groups relating to PrEP in their country. Private providers were asked about their organization's role in delivering OC/PrEP, the typical OC/PrEP client profile, challenges and solutions to OC/PrEP delivery, interest in delivering the DPP, and potential opportunities for and challenges to delivering the DPP in their setting. Interviews took place in two rounds, with 85 interviews conducted in April and May 2022, and a second round of 21 interviews conducted from October to December 2022. The second round placed a stronger focus on evidence gaps identified from the first round, such as information on subsidy models and willingness‐to‐pay, with interviews targeted at key individuals and partners that could provide the most strategic thinking, as well as those who could be potential implementers in the rollout of the DPP.

### 2.3 Stakeholder consultations

Stakeholder consultation meetings were held in Nairobi, Kenya, in January 2023 and Harare, Zimbabwe, in February 2023. The objectives of the meetings were to review and provide feedback on the findings to date, agree on the recommended private sector channels and proposed phasing for DPP introduction, and identify key partners and actions to consider in preparation for rollout. These consultation meetings included participants from private sector channels, implementing partners, medical professional bodies and representatives from Ministries of Health (MoH). In Kenya, a single consultation meeting was held. In Zimbabwe, three separate consultations were held: one with networked private providers, including pharmacies; a second with implementing partners, academics and donors; and a third with advocacy groups and members of the AVAC Advocacy Fellows Programme, a network of HIV prevention advocates. No consultation meetings were held in South Africa following feedback from local stakeholders that country‐level engagement on the DPP should only be held after the DPP's pilot bioequivalence study was successfully completed.

### 2.4 Analysis

Data collected during the desk review, KIIs and stakeholder consultations were analysed thematically by the research team to assess the potential of 10 private sector delivery channels identified through the desk review and national scoping exercises (Table 1), noting that channel designations may overlap, complement or be dependent upon one another. One senior researcher reviewed and synthesized the themes identified by the research team to draw key insights from the data and probe deeper where needed. Channels were evaluated against eight key market criteria (Table 3). For each criterion, channels were scored as high performance, average performance or low performance. Based on these ratings, each channel was assigned one of three designations: (1) currently recommended for delivery of the DPP; (2) has potential for DPP delivery but not currently recommended; and (3) does not currently have potential for DPP delivery. To be designated as recommended for the delivery of the DPP, a channel needed to either have scored as high performance under policy and regulation, with no more than one low‐performance criteria or have scored as high performance under scalability, sustainability or geographical coverage, with no more than one low‐performance criteria. Exceptions were made for newer and rapidly growing private sector channels such as telehealth and telemedicine, given that they demonstrate significant potential but currently have little data or evidence on the market criteria.

**Table 3:** Market criteria for channel analysis

Market criteria	Description
**Policy and regulation**	The formal policies, strategies, plans, regulations and guidelines that are in place to support the effective delivery of PrEP and OC, the DPP and other MPTs.
**Public‐private coordination**	The extent to which the public and private sector actively and effectively plan and coordinate their activities to support the delivery of PrEP and OC, and the extent to which they might coordinate around the DPP and MPT delivery.
**Monitoring and evaluation**	The extent to which market data on PrEP and OC is currently being monitored, tracked and available, and subsequently shared and used, to improve overall delivery.
**Supply chain**	The availability of PrEP and OC through the public and private sectors, and the overall strength and reliability of the country's supply chain system.
**Financing**	The extent to which the DPP will need to be subsidized in order for demand and use of the product to be significant.
**Scalability**	The extent to which the delivery channel could be scaled, depending on a range of factors, including the pricing of product, continued support from donors and the extent to which markets remain balanced and healthy.
**Sustainability**	To what degree the channel is or might be financially sustainable in the future, depending on a range of factors, including the pricing of the product, continued support from donors, the extent to which markets remain balanced and healthy, and increased and maintained demand for products.
**Geographic coverage**	The extent to which the channel reaches across the country, including urban and rural locations.

### 2.5 Ethical approval

Approval from an Ethics Review Board was not sought for this applied research, which did not collect data on human participants. Data collection was restricted to stakeholder views on service delivery and policy environments and did not include respondents’ personal information.

## 3 RESULTS

**Figure 1:**
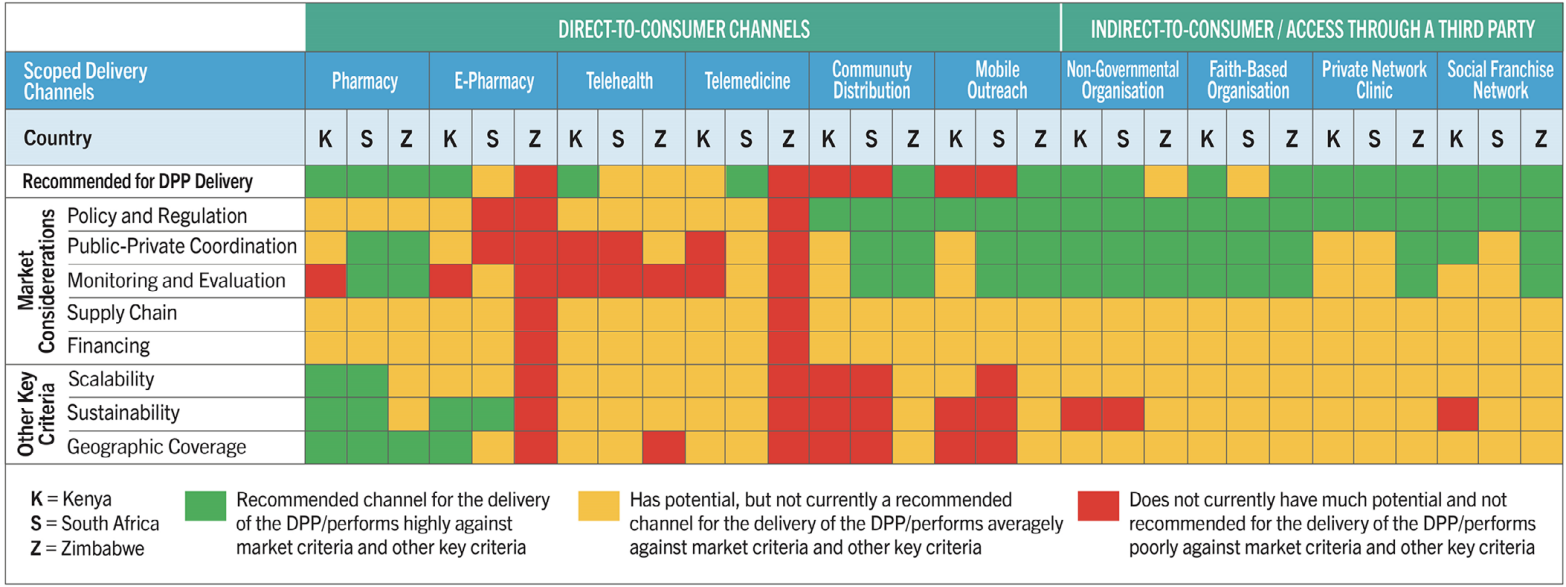
Market criteria for channel analysis.

**Figure 2:**
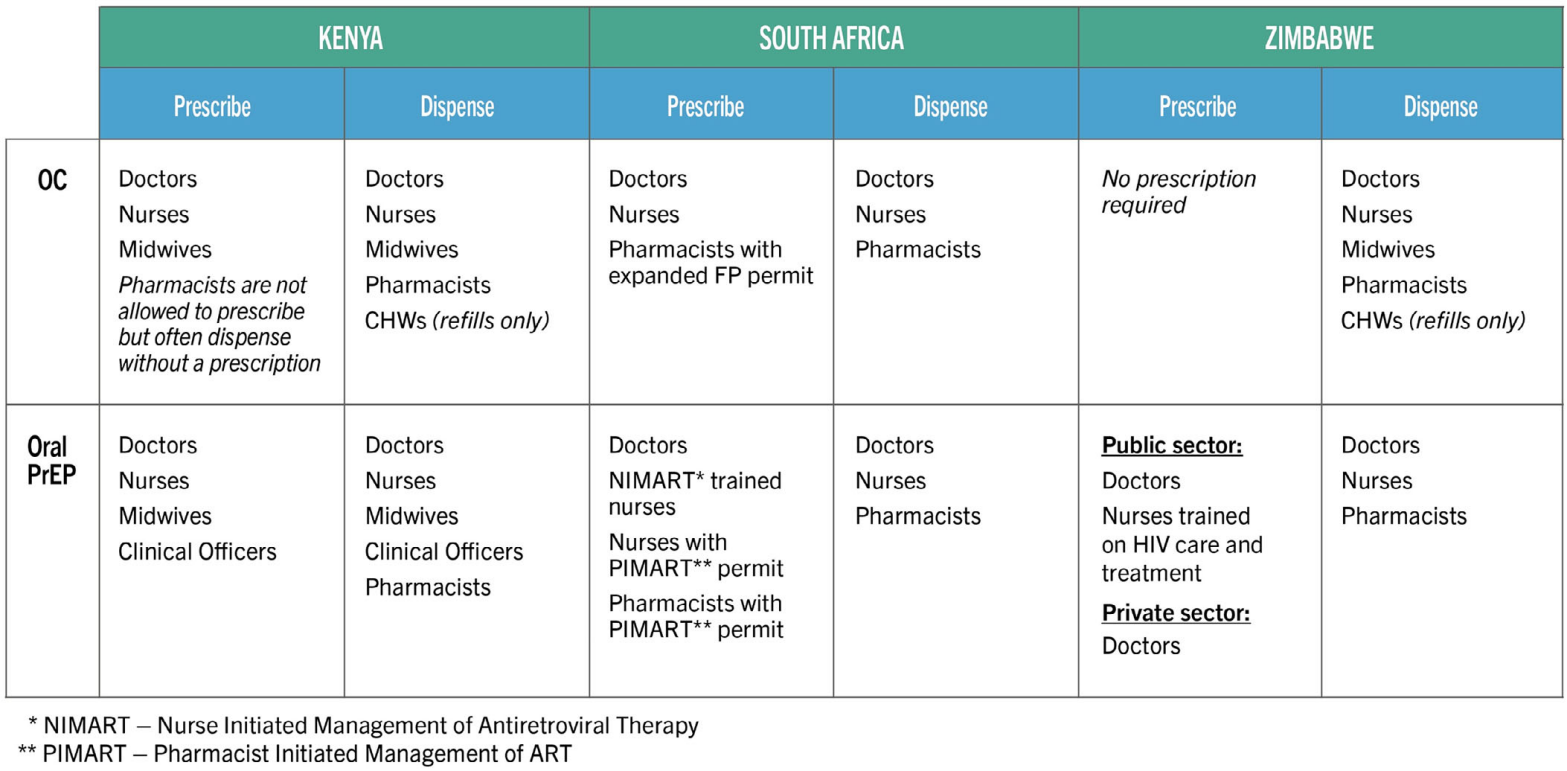
Summary of cadres’ ability to prescribe and dispense OC and PrEP by country.

Figure 1 provides a summary of how each channel scored against eight market criteria, along with the channel's overall rating. Pharmacies and networked private providers performed highly across all three countries. In Kenya, e‐pharmacies and telehealth showed high potential, along with telemedicine in South Africa and public‐private partnerships (PPPs) in Zimbabwe. Table 4 shows the proposed phasing for DPP rollout across the recommended channels, based on channel readiness to deliver both OC and PrEP, with the assumption that the DPP is commercially available in all three countries starting in 2025. A significant factor in a channel's potential to provide the DPP in the near term is national regulation on which cadres can provide both OC and PrEP, which is summarized in Figure 2.

**Table 4:** Proposed channel phasing for DPP rollout

Country	Phasing	Channels
**Kenya**	Phase 1: 2025−2026	Private provider networks, including social franchises and clinics run by NGOs and FBOs Pharmacies, with links to other providers for prescribing E‐pharmacies, with links to other providers for prescribing
	Phase 2: 2027−2029	Pharmacies, for both prescribing and dispensing E‐pharmacies, for both prescribing and dispensing Telehealth
**South Africa**	Phase 1: 2025−2026	Private provider networks
	Phase 2: 2027−2029	Pharmacies Telemedicine
**Zimbabwe**	Phase 1: 2025−2026	Private provider networks Pharmacies, with links to other providers for prescribing
	Phase 2: 2027−2029	Pharmacies, for both prescribing and dispensing Public‐private partnerships

### 3.1 Kenya

Private provider networks, including social franchises and clinics run by Non‐governmental organisations (NGOs) and Faith‐based organisations (FBOs), are well‐established providers of FP and HIV services, making them a priority for Phase 1 DPP rollout, though they are less engaged with HIV service provision, and generally focus on long‐acting FP methods over short‐acting methods like OC. These private providers have wide geographic reach, with approximately 600 franchised facilities nationwide, and large FBO networks in rural areas. Prices may be lower than other private providers, but generally include a consultation fee on top of the commodity price, making this channel more expensive than pharmacies. All necessary policies and regulations are already in place for PrEP and OC provision in these facilities, including PrEP initiation, and M&E systems are generally strong, often with robust linkages to MoH systems. Additional delivery channels, such as community health workers, mobile outreach and telemedicine apps, are frequently integrated into private provider networks, providing an opportunity to serve clients outside of facilities and distribute refills.

With nearly 7000 registered outlets nationwide [23], covering both urban and rural areas, pharmacies are frequently clients’ first point of contact when confronted with an illness [24] due to the fact they are discreet, convenient and fast. Pharmacies provide services to clients from a wide range of socio‐economic backgrounds, including the poorest [25], and are perceived as being more anonymous and less stigmatizing than private providers. This perception makes pharmacies a popular choice for sexual and reproductive health (SRH) and HIV services, including OC and PrEP. While pharmacies are currently only authorized to dispense PrEP, rather than initiate it, an ongoing pilot study, the Pharm PrEP Trial, funded by the Gates Foundation and run by the Kenya Medical Research Institute; Jomo Kenyatta University of Agriculture and Technology; Fred Hutchinson Cancer Center; and Jhpiego, has shown that pharmacists can be trained to deliver high‐quality PrEP services, including initiations [26], and both the National AIDS and STIs Control Programme (NASCOP) and the Pharmacy and Poisons Board have expressed strong support for this model, resulting in the development of a care pathway for pharmacy‐based delivery of PrEP [27]. Considering both their popularity and current national regulations, pharmacies with links to channels which can prescribe PrEP, such as private providers or telehealth platforms, are prioritized for Phase 1 DPP rollout, while pharmacist‐initiated DPP, if approved, is prioritized for Phase 2.

E‐pharmacies, whose lack of face‐to‐face engagement provides an additional layer of discretion over traditional pharmacies, are a growing entry point for SRH and HIV services and commodities, including OC and PrEP. Accessible online 24 hours per day, and with longer opening hours for virtual consultations than traditional private providers, they also offer convenience. Though e‐pharmacies are currently concentrated in urban locations, they are rapidly expanding and increasing coverage of their delivery areas; however, the use of e‐pharmacies is still limited to those with internet access—estimated in 2022 as 42% of the population [28]. Like pharmacies, e‐pharmacies can only dispense PrEP, though a Gates Foundation‐funded pilot study, the ePrEP Kenya Pilot, will explore PrEP and post‐exposure prophylaxis (PEP) delivery, including initiation, via the e‐pharmacy MYDAWA. Participants will receive PrEP or PEP following a telehealth consultation with a clinician to assess PrEP/PEP eligibility and an HIV test to confirm negative status [29]. MYDAWA currently sells 25% of all HIV self‐testing kits in Kenya, demonstrating a strong demand for HIV services through an e‐pharmacy platform. The rapid growth of this channel makes it a priority for Phase 1 rollout for dispensing the DPP, and a Phase 2 priority for initiation if approved.

Telehealth, which includes telemedicine, digital demand creation and remote counselling, is another fast‐growing channel, presently encompassing around 40 providers with country‐wide reach. Like e‐pharmacies, they offer clients an extra layer of both discretion and convenience, with longer opening hours than static facilities, including some who operate 24 hours per day. Although most telehealth providers do not yet have dispensing capabilities, due to the fact the national and county eHealth bills which regulate this channel were only passed shortly after this analysis was completed [30], they experience high demand for SRH and HIV services from clients, whom they refer onwards to pharmacies or e‐pharmacies. The inability of most providers to currently dispense medications makes this channel a Phase 2 priority. Telehealth apps also support both clients and providers with tools to support the client journey, including chatbots, counselling tools and linkages to services, which can be an important add‐on to other channels, particularly as telehealth apps are sometimes the first point‐of‐contact a client has with the health system before taking up SRH or HIV services.

### 3.2 South Africa

Private provider networks, such as commercial clinic chains, general practitioner (GP) networks and networks supported by the South African HIV Clinicians Society, comprise up to 3500 providers nationwide and serve about 29% of the population. They have a long history of providing both FP and HIV services, so are prioritized for Phase 1 rollout, though have been slower to roll out PrEP, citing a lack of effective demand generation and provider knowledge. Provider networks advocated strongly for the inclusion of PrEP in private health insurance packages, though 3 years after adding PrEP, one of the country's largest insurers reported only 18 PrEP users. Clients eligible for National Health Insurance can access PrEP free of charge from private providers under a pilot initiative called GP Care Cell, a network of private GPs contracted to provide HIV and FP services on behalf of the National Department of Health (NDoH) using NDoH commodities. With over 50 GPs and eight community pharmacies participating as of 2023, the initiative has demonstrated that private providers can manage government commodities effectively and efficiently [31].

Pharmacies have wide geographic coverage, with over 4700 outlets nationwide [32], covering both urban and rural locations. They are often the first point‐of‐call for clients seeking healthcare, though they are a less popular channel for PrEP or OC services, which are overwhelmingly accessed via the public sector [16, 33]. Historically, pharmacies have been legally permitted to dispense but not initiate both PrEP and OC. In 2019, the government introduced Pharmacist Initiated Management of Antiretroviral Therapy (PIMART), an expanded scope of practice allowing pharmacists who have completed additional training and received a permit to initiate clients on ARV medications, including PrEP. PIMART also expands pharmacists’ scope of practice for FP provision, allowing the initiation of several FP methods, including OC. As of 2023, more than 1000 pharmacists had received PIMART training, with over 300 of these currently initiating clients on PrEP. Although a 2021 court case brought by GPs placed the rollout of PIMART on hold, the Pretoria High Court's decision in August 2023 to dismiss the case has allowed the rollout to resume [34]. Several pilot studies are taking place to explore safety, efficiency and willingness‐to‐pay for PrEP service delivery under PIMART. The fact that the hold placed on PIMART has only recently been removed makes pharmacies a Phase 2 priority.

The telemedicine sector experienced strong growth in conjunction with the COVID‐19 pandemic, and with smartphone penetration estimated at 90% nationwide [35], it has significant potential to grow further, though data on current telemedicine market size and reach is not available. Telemedicine offers discretion, with virtual clinician consultations, and convenience, with longer opening times than traditional channels, and is accessible nationwide. Telemedicine is also used within primary care facilities to connect a patient to a doctor or specialist during a nurse consultation. There are several online telemedicine platforms specializing in SRH and women's health, including some which dispense OC, although none currently offer PrEP. OC is dispensed either via a courier or as a prescription which a client must take to a pharmacy. Current regulations require laboratory‐based hepatitis B and creatinine screening for PrEP initiations, potentially making PrEP access via telemedicine more complex, although these requirements are expected to change now that the PIMART hold has been lifted. This makes telemedicine a Phase 2 priority.

### 3.3 Zimbabwe

Networked private providers, including social franchises and facilities run by NGOs and FBOs, are a priority for Phase 1 DPP rollout, due to being a popular entry point for FP and HIV services, and having a wide geographic reach, with over 800 facilities in low‐income high‐density urban areas and rural areas. They offer both OC and PrEP, though to date PrEP provision has been limited, and focussed on key populations. The networks have well‐established, organized channels for commodity distribution, with structures and systems in place to efficiently roll out new products. Many also have memoranda of understanding with the Ministry of Health and Childcare (MoHCC) to receive and distribute government‐funded commodities, helping to keep services affordable. Commercial franchises, which are more expensive than other networked providers, but generally less expensive than stand‐alone facilities, have been growing in popularity among those with medical insurance or who can afford user fees. Primarily located in urban and peri‐urban settings, their higher price point is associated with improved quality of care.

The pharmacy channel has a wide reach, with over 900 outlets nationwide [36], though coverage is concentrated in urban areas. Pharmacies are a popular source of OC; pharmacists’ ability to dispense OC without a prescription, government‐subsidized prices and lack of consultation fees make this a convenient and affordable option for many. Under current regulations, pharmacists can dispense but not initiate clients on PrEP. The Community Pharmacies Association is engaged with the MoHCC on task‐shifting HIV service provision, including PrEP initiations, to pharmacists as part of the government's objective to expand access to comprehensive, quality HIV and tuberculosis (TB) services through effective PPPs. This includes strengthening their capacity to build, promote and sustain partnerships within the Strategic Framework for TB/HIV Prevention, Treatment, Care, and Support Public‐Private‐Partnership, 2021–2025. Pharmacists’ current inability to initiate clients on PrEP makes this channel a Phase 1 priority for dispensing the DPP and a Phase 2 priority for full initiation.

Though not a standalone delivery channel, PPP models, which support private sector channels by strengthening linkages between public and private providers to generate demand and uptake for healthcare services, are increasingly recognized by donors and NGOs as critical for the impactful delivery of HIV services, particularly in the case of supply chains. Formal PPPs, governed by the TB/HIV PPP Strategic Framework, will be necessary to allow most private sector channels to distribute MoHCC commodities. The strategies within the framework can provide useful models for effective systems of private sector engagement, and the introduction of systems to ensure safeguards of public commodities. Implementers funded by the U.S. President's Emergency Plan for AIDS Relief (PEPFAR) are integrating PPP capacity building and service integration into objectives for public sector and community‐based programmes. Leveraging PPPs for DPP delivery is a Phase 2 priority to allow time for the TB/HIV PPP Strategic Framework to be fully implemented.

## 4 DISCUSSION

Assessing 10 private sector delivery channels across three countries for potential to deliver the DPP based on policies, level of coordination with the public sector, data systems, supply chain, subsidy need, scalability, sustainability and geographic coverage, we found the most promising channels were social franchises and NBO/FBO‐run facilities, pharmacies, e‐pharmacies and telehealth platforms in Kenya; private provider networks, pharmacies and telemedicine in South Africa; and social and commercial franchises, pharmacies and PPP models in Zimbabwe.

One of our principal findings was that while a significant proportion of FP services in Kenya (22%) [8], South Africa (11.4%) [16] and Zimbabwe (17.3%) [8] are delivered via the private sector, it is still an untapped delivery channel for PrEP. Addressing the underlying reasons why this is the case will be a prerequisite to DPP rollout, not just in these three countries, but in all countries with high HIV incidence where the private sector is a popular source for FP, such as Eswatini, Lesotho, Malawi, Namibia, Uganda and Zambia [2, 8]. While among well‐established channels, low levels of PrEP delivery may be due to a knowledge and support gap, in newer channels such as pharmacies and telehealth, the primary barrier is regulation. Increasing private sector delivery of PrEP thus requires a two‐pronged approach. The first involves engaging government regulators, MoH and professional bodies on updating PrEP delivery regulations, including regarding which cadres can deliver PrEP and where, to bring them in line with those for OC. Task‐shifting PrEP delivery would not only increase the number of PrEP access points, but could reach populations not already engaged in care that could benefit from PrEP [37]. Evidence has shown this to be an effective method of increasing access to both HIV [38] and FP services [39]. The second is utilizing proven strategies from social franchising to engage providers, both traditional and technology‐based, on the delivery of HIV prevention services, including supporting them with supply‐side interventions such as training, quality assurance and access to affordable commodities, and demand‐side interventions such as marketing support [40].

Ensuring policies are in place to allow delivery of PrEP and OC by the same provider in the same location can facilitate the integration of FP and HIV prevention services, increasing access to both, and would prepare a pathway for future MPTs [41, 42]. As new PrEP options come to market [5], and FP implementers begin to integrate PrEP [43], providers can leverage experience with choice‐based FP counselling to support clients to identify the HIV prevention method which is right for them, and apply this knowledge to MPTs like the DPP when available [44]. In addition, promoting integration at a system level, such as for government reporting or supply chain management, where FP and HIV services are often siloed [41], can simplify processes for providers and the government, saving time and money, and potentially improving data quality or reducing stock‐outs.

Another important finding was that newer, technology‐based channels are uniquely suited to deliver services associated with a high level of stigma, such as HIV or FP services. The focus on the delivery of oral PrEP via HIV clinics during the initial rollout phase has been cited as a factor in low uptake [45], partially due to the stigma associated with these facilities. Lack of face‐to‐face contact and a higher level of discretion than brick‐and‐mortar facilities may help make technology‐based channels the preferred option for users facing discrimination in accessing healthcare services, such as adolescents, sex workers and members of the lesbian, gay, bisexual, transgender, and queer (LGBTQ+) community, all of whom are key populations for HIV services [46]. Robust regulation allowing PrEP and FP delivery via these channels would not only increase access to these services, but may also increase access specifically among those who could most benefit.

A major unanswered question from our study is how price and willingness‐to‐pay could impact the rollout of the DPP within the private sector. Though the proposed price of the DPP in the three study countries is unknown at this time, OC, which costs $0.50−$26 USD per month, tends to be much less expensive than PrEP, which costs $20−$80 USD per month; therefore, it is likely to be the PrEP component which drives the price. Though data on willingness‐to‐pay for PrEP in the private sector is scant, evidence from Kenya shows that women are willing to pay for pharmacy‐delivered PrEP services at a median price of $2.71 USD per month [47], significantly less than the full retail price range. Further data from Nigeria also suggest that while potential PrEP users are willing to pay for PrEP, there is a gap between what they are willing to pay and current retail prices [48]. For FP services, evidence has shown that the private sector is most commonly accessed by clients in the top wealth quintile [49], suggesting that only the wealthiest are willing, or able, to pay private sector prices for FP. This is a strong indication that the DPP is likely to require a subsidy to reach potential users through the private sector, though further research on both willingness and ability‐to‐pay for the DPP is needed. Due to the diverse nature of the private sector, consideration will need to be given to the level and type of incentives needed by channel, as this will vary with the profile of the clients most likely to use each channel. In addition, as most subsidized PrEP supplies enter high HIV prevalence countries as donated commodities distributed by governments, PPPs may become an important component of private sector PrEP delivery, though the use of such commodities will require initial engagement with the MoH to determine distribution and reporting requirements.

Our study had a number of strengths, including direct consultation with government stakeholders, who have an intimate understanding of government health priorities and policy, and with representatives from a wide variety of private sector channels with direct experience delivering OC and/or PrEP. In addition, our study team included HIV and SRH technical experts from the study countries who each have over 15 years’ experience working on HIV prevention and FP programmes and strong working relationships with both governments and private sector providers. This enabled the team to identify and reach the most relevant individuals and organizations for consultations and to acquire the key information required. Our study also had several limitations. A major limitation was a lack of data on the pricing of commodities, how this is determined and clients’ willingness‐to‐pay for PrEP. As the success of the DPP in the private sector will rely not only on accessibility, but also price, having data on how commodities are priced is paramount when assessing potential service delivery channels. This missing information limits our ability to draw conclusions on the potential role of the private sector in DPP delivery. An additional limitation was incomplete data on the uptake of FP and HIV services in the private sector, especially the newer, technology‐based channels, due to a combination of concerns over sharing business intelligence and data not being available. Without these data, it is difficult to make direct comparisons between channels on where FP and HIV prevention uptake is highest. Our study was also limited by the fact the literature review only included materials in English. Despite these limitations, our data show high potential for the delivery of the DPP via private sector channels, and a summary of recommended actions to take this forward is included in Table 5.

**Table 5:** Recommended actions to facilitate the private sector introduction of the DPP in Kenya, South Africa and Zimbabwe

Intervention type	Recommendation
**Policy and regulation**	Engage government regulators, MoH and professional bodies on updating PrEP delivery regulations, including who can deliver PrEP and via which channels, leveraging evidence generated by studies such as the Pharm PrEP Trial and ePrEP Kenya Pilot.
**Policy and regulation**	Engage MoH to create opportunities for PPPs where appropriate and to determine supply chain, distribution and reporting requirements for private sector channels to access the DPP through government supplies and to report on usage and uptake.
**Demand side**	Support private providers with marketing materials and strategies.
**Demand side**	Conduct a willingness‐to‐pay study across different potential client groups (e.g. rural/urban) and private sector channels.
**Supply side**	Support private providers with training, quality assurance and access to commodities at a price which will allow them to generate a profit.
**Supply side**	Conduct a costing study across different private sector channels to determine additional subsidies or payments that may be required to ensure consultation fees or user fees are within the levels of willingness‐to‐pay.

## 5 CONCLUSIONS

The private sector landscapes in Kenya, South Africa and Zimbabwe are well‐suited for the introduction of the DPP, particularly via pharmacies, networked private providers, and newer, technology‐based channels such as e‐pharmacies and telemedicine, with country‐specific differences elaborated on throughout this manuscript. Successful DPP rollout will depend on a number of factors, including the level of provider engagement with the delivery of HIV prevention services and where and by whom local regulation allows PrEP and OC to be delivered. The integration of FP and HIV services can both improve access to these services and create a pathway for the introduction of the DPP and other MPTs. Including newer, technology‐based channels, which offer increased discretion, in DPP rollout plans could increase the uptake of the DPP among populations with a need for contraception and HIV prevention. Further research is needed on willingness‐to‐pay to understand the impact of pricing on private sector DPP delivery.

## COMPETING INTERESTS

We declare no competing interests.

## AUTHORS’ CONTRIBUTIONS

CVH drafted the manuscript. EL, RW, TB, TC and KW performed data collection and analysis, drafted a report of the findings and reviewed the manuscript. KS reviewed the manuscript.

## FUNDING

This applied research was supported by generous grant #2012‐05668 from the Children's Investment Fund Foundation (CIFF) to AVAC.

## DISCLAIMER

The views expressed are those of the authors, and the contents do not necessarily reflect the views of CIFF.

## Supporting information

Supporting Information file 1: List of Desk Review Search Terms
A document with a list of search terms used for the desk review portion of the methodology.

## Data Availability

Research data are not shared.
