# Supplementary material for: Harnessing private sector strategies for family planning to deliver the Dual Prevention Pill, the first multipurpose prevention technology with pre‐exposure prophylaxis, in an expanding HIV prevention landscape
Source: J Int AIDS Soc. 2024 Aug 15;27(8):e26346. doi: 10.1002/jia2.26346 (PMC11327274; doi:10.1002/jia2.26346)
Supplement: Supplementary file 1 — Supporting Information file 1: List of Desk Review Search Terms A document with a list of search terms used for the desk review portion of the methodology. [file JIA2-27-e26346-s001.docx]

**Supporting Information 1- List of Desk Review Search Terms**

PrEP private sector

PrEP Kenya

PrEP South Africa

PrEP Zimbabwe

PrEP pharmacy

PrEP private clinic

PrEP physician

PrEP private physician

PrEP doctor

PrEP private doctor

PrEP private health centre

PrEP telemedicine

PrEP online

PrEP FBO

PrEP private medical provider

PrEP private nurse

PrEP social marketing

PrEP social franchise

PrEP drug shop

PrEP PIMART

FP private sector

FP Kenya

FP South Africa

FP Zimbabwe

FP pharmacy

FP private clinic

FP physician

FP private physician

FP doctor

FP private doctor

FP private health centre

FP telemedicine

FP online

FP FBO

FP private medical provider

FP private nurse

FP social marketing

FP social franchise

FP drug shop

FP PIMART

Oral contraceptive private sector

Oral contraceptive Kenya

Oral contraceptive South Africa

Oral contraceptive Zimbabwe

Oral contraceptive pharmacy

Oral contraceptive private clinic

Oral contraceptive physician

Oral contraceptive private physician

Oral contraceptive doctor

Oral contraceptive private doctor

Oral contraceptive private health centre

Oral contraceptive telemedicine

Oral contraceptive online

Oral contraceptive FBO

Oral contraceptive private medical provider

Oral contraceptive private nurse

Oral contraceptive social marketing

Oral contraceptive social franchise

Oral contraceptive drug shop

Oral contraceptive PIMART

COC private sector

COC Kenya

COC South Africa

COC Zimbabwe

COC pharmacy

COC private clinic

COC physician

COC private physician

COC doctor

COC private doctor

COC private health centre

COC telemedicine

COC online

COC FBO

COC private medical provider

COC private nurse

COC social marketing

COC social franchise

COC drug shop

COC PIMART

Contraceptive private sector

Contraceptive Kenya

Contraceptive South Africa

Contraceptive Zimbabwe

Contraceptive pharmacy

Contraceptive private clinic

Contraceptive physician

Contraceptive private physician

Contraceptive doctor

Contraceptive private doctor

Contraceptive private health centre

Contraceptive telemedicine

Contraceptive online

Contraceptive FBO

Contraceptive private medical provider

Contraceptive private nurse

Contraceptive social marketing

Contraceptive social franchise

Contraceptive drug shop

Contraceptive PIMART

Daily pill private sector

Daily pill Kenya

Daily pill South Africa

Daily pill Zimbabwe

Daily pill pharmacy

Daily pill private clinic

Daily pill physician

Daily pill private physician

Daily pill doctor

Daily pill private doctor

Daily pill private health centre

Daily pill telemedicine

Daily pill online

Daily pill FBO

Daily pill private medical provider

Daily pill private nurse

Daily pill social marketing

Daily pill social franchise

Daily pill drug shop

Daily pill PIMART

“The pill” private sector

“The pill” Kenya

“The pill” South Africa

“The pill” Zimbabwe

“The pill” pharmacy

“The pill” private clinic

“The pill” physician

“The pill” private physician

“The pill” doctor

“The pill” private doctor

“The pill” private health centre

“The pill” telemedicine

“The pill” online

“The pill” FBO

“The pill” private medical provider

“The pill” private nurse

“The pill” social marketing

“The pill” social franchise

“The pill” drug shop

“The pill” PIMART

Emergency contraception private sector

Emergency contraception Kenya

Emergency contraception South Africa

Emergency contraception Zimbabwe

Emergency contraception pharmacy

Emergency contraception private clinic

Emergency contraception physician

Emergency contraception private physician

Emergency contraception doctor

Emergency contraception private doctor

Emergency contraception private health centre

Emergency contraception telemedicine

Emergency contraception online

Emergency contraception FBO

Emergency contraception private medical provider

Emergency contraception private nurse

Emergency contraception social marketing

Emergency contraception social franchise

Emergency contraception drug shop

Emergency contraception PIMART

Injectable contraception private sector

Injectable contraception Kenya

Injectable contraception South Africa

Injectable contraception Zimbabwe

Injectable contraception pharmacy

Injectable contraception private clinic

Injectable contraception physician

Injectable contraception private physician

Injectable contraception doctor

Injectable contraception private doctor

Injectable contraception private health centre

Injectable contraception telemedicine

Injectable contraception online

Injectable contraception FBO

Injectable contraception private medical provider

Injectable contraception private nurse

Injectable contraception social marketing

Injectable contraception social franchise

Injectable contraception drug shop

Injectable contraception PIMART

LARC private sector

LARC Kenya

LARC South Africa

LARC Zimbabwe

LARC pharmacy

LARC private clinic

LARC physician

LARC private physician

LARC doctor

LARC private doctor

LARC private health centre

LARC telemedicine

LARC online

LARC FBO

LARC private medical provider

LARC private nurse

LARC social marketing

LARC social franchise

LARC drug shop

LARC PIMART

Short-acting contraception private sector

Short-acting contraception Kenya

Short-acting contraception South Africa

Short-acting contraception Zimbabwe

Short-acting contraception pharmacy

Short-acting contraception private clinic

Short-acting contraception physician

Short-acting contraception private physician

Short-acting contraception doctor

Short-acting contraception private doctor

Short-acting contraception private health centre

Short-acting contraception telemedicine

Short-acting contraception online

Short-acting contraception FBO

Short-acting contraception private medical provider

Short-acting contraception private nurse

Short-acting contraception social marketing

Short-acting contraception social franchise

Short-acting contraception drug shop

Short-acting contraception PIMART

FP public private partnership

PrEP public private partnership

FP total market approach

PrEP total market approach
